# Polygenic Scores Predict Alcohol Problems in an Independent Sample and Show Moderation by the Environment

**DOI:** 10.3390/genes5020330

**Published:** 2014-04-10

**Authors:** Jessica E. Salvatore, Fazil Aliev, Alexis C. Edwards, David M. Evans, John Macleod, Matthew Hickman, Glyn Lewis, Kenneth S. Kendler, Anu Loukola, Tellervo Korhonen, Antti Latvala, Richard J. Rose, Jaakko Kaprio, Danielle M. Dick

**Affiliations:** 1 Department of Psychiatry, Virginia Commonwealth University, P.O. Box 980126, Richmond, VA 23298, USA; E-Mails: faliev@vcu.edu (F.A.); edwards.alexis@gmail.com (A.C.E.); kendler@vcu.edu (K.S.K.); 2 School of Social and Community Medicine, University of Bristol, Canynge Hall, 39 Whatley Road, Bristol, BS8 2PS, UK; E-Mails: Dave.Evans@bristol.ac.uk (D.M.E.); john.macleod@bristol.ac.uk (J.M.); matthew.hickman@bristol.ac.uk (M.H.); 3 Translational Research Institute, University of Queensland, Level 7, 37 Kent Street, Woolloongabba, Brisbane QLD 4102, Queensland, Australia; 4 Diamantina Institute, University of Queensland, Level 7, 37 Kent Street, Woolloongabba, Brisbane QLD 4102, Queensland, Australia; 5 Division of Psychiatry, University College London, 67-73 Riding House St., London W1W 7EJ, UK; E-Mail: glyn.lewis@ucl.ac.uk; 6 Department of Public Health, Hjelt Institute, University of Helsinki, P.O. Box 41, Helsinki FI-00014, Finland; E-Mails: anu.loukola@helsinki.fi (A.Lo.); tellervo.korhonen@helsinki.fi (T.K.); antti.latvala@helsinki.fi (A.La.); jaakko.kaprio@helsinki.fi (J.K.); 7 National Institute for Health and Welfare, Department of Mental Health and Substance Abuse Services, P.O. Box 30, Mannerheimintie 166, Helsinki FI-00300, Finland; 8 Department of Psychological and Brain Sciences, Indiana University, 1101 East 10th St., Bloomington, IN 47405, USA; E-Mail: rose@indiana.edu; 9 University of Helsinki, Institute for Molecular Medicine (FIMM), P.O. Box 20, Tukholmankatu 8, Helsinki FI-00014, Finland

**Keywords:** alcohol problems, adolescence, gene-by-environment, ALSPAC, FinnTwin12

## Abstract

Alcohol problems represent a classic example of a complex behavioral outcome that is likely influenced by many genes of small effect. A polygenic approach, which examines aggregate measured genetic effects, can have predictive power in cases where individual genes or genetic variants do not. In the current study, we first tested whether polygenic risk for alcohol problems—derived from genome-wide association estimates of an alcohol problems factor score from the age 18 assessment of the Avon Longitudinal Study of Parents and Children (ALSPAC; n = 4304 individuals of European descent; 57% female)—predicted alcohol problems earlier in development (age 14) in an independent sample (FinnTwin12; n = 1162; 53% female). We then tested whether environmental factors (parental knowledge and peer deviance) moderated polygenic risk to predict alcohol problems in the FinnTwin12 sample. We found evidence for both polygenic association and for additive polygene-environment interaction. Higher polygenic scores predicted a greater number of alcohol problems (range of Pearson partial correlations 0.07–0.08, all *p-*values ≤ 0.01). Moreover, genetic influences were significantly more pronounced under conditions of low parental knowledge or high peer deviance (unstandardized regression coefficients (*b*), *p*-values (*p*), and percent of variance (*R*^2^) accounted for by interaction terms: *b* = 1.54, *p* = 0.02, *R*^2^ = 0.33%; *b* = 0.94, *p* = 0.04, *R*^2^ = 0.30%, respectively). Supplementary set-based analyses indicated that the individual top single nucleotide polymorphisms (SNPs) contributing to the polygenic scores were not individually enriched for gene-environment interaction. Although the magnitude of the observed effects are small, this study illustrates the usefulness of polygenic approaches for understanding the pathways by which measured genetic predispositions come together with environmental factors to predict complex behavioral outcomes.

## 1. Introduction

Alcohol consumption and related problems are classic examples of complex behavioral outcomes that likely involve many genes of small effect [1]. Twin studies, which infer genetic influences by comparing the phenotypic similarity between monozygotic (MZ) twins (who share all of their genetic variation) and dizygotic (DZ) twins (who share half of their genetic variation, on average), have been crucial for demonstrating that latent genetic influences account for a considerable amount of the variation in measures of alcohol consumption and problems, with heritability estimates in the range of 50%–60% [2,3,4,5]. Twin studies have also been critical for demonstrating that environmental factors moderate the importance of genetic influences. In adolescents, for example, genetic influences on alcohol use and other closely related externalizing problems (e.g., conduct problems) increase under conditions of low parental knowledge (*i.e.*, the degree to which parents know about one’s daily activities and associates) or high peer deviance (*i.e.*, the degree to which one’s peer group engages in substance use and antisocial behavior) [6,7,8,9]. Thus, genetic influences appear to become more important under environmental conditions characterized by more social opportunity and less social control [10]. 

In contrast to the consistent evidence for the heritability of alcohol use and problems, no robust associations have been detected in genome-wide association studies (GWAS) to date. This is the case, in part, because the small samples typically used in alcohol research are underpowered to detect the very modest individual effect sizes that are generally observed in GWAS of complex behavioral outcomes. Large meta- and mega-analyses pooling across many studies are needed to obtain robust results in the substance use area [11]; only now are these studies underway for alcohol use and alcohol problems. In candidate gene studies, a few compelling associations have emerged within biologically plausible pathways. For example, polymorphisms in *ADH1B* and *ALDH2* genes, which code for alcohol-metabolizing enzymes, have well-replicated associations with alcohol dependence [12,13,14,15]. In another example, independent groups have found evidence that the α2 encoding subunit of the GABA-A receptor (*GABRA2*) is associated with alcohol dependence [16,17]. Likewise, despite consistent evidence from twin samples that environmental factors moderate latent genetic influences, measured gene-by-environment moderation effects for behavioral outcomes have been widely criticized on the grounds that they are underpowered and likely reflect Type I statistical error [18]. 

In the absence of success in identifying individual genes that account for a substantial proportion of the variance in alcohol outcomes, and lack of expectation that such genes will be found in the near future, polygenic approaches have emerged as one paradigm for examining aggregate measured genetic effects that can have predictive power when individual genes cannot [19]. This approach typically uses results from a genome-wide association study in a discovery sample. Using a *p*-value threshold much more liberal than what would be required for genome-wide significance, a polygenic risk score for each individual in an independent target sample is calculated by summing up the number of alleles for each single nucleotide polymorphism (SNP) weighted by the effect size drawn from a GWAS. The score then represents the composite additive effect of these multiple variants, which likely includes a mixture of true genetic signals and noise. 

In the current study, we adopted a polygenic approach to examine alcohol problems in adolescence. Adolescence represents an important developmental period for the initiation of alcohol use [20], and, for some, the development of alcohol problems [21]. Longitudinal developmental studies indicate that the heritability of alcohol use increases across adolescence [4,22], making this an important period of the lifespan for beginning to identify the genetic predispositions toward alcohol problems, and how these predispositions interface with key environmental factors (e.g., low parental knowledge and affiliations with deviant peers) known to be associated with higher levels of alcohol problems. We tested the hypotheses that: (1) polygenic risk for alcohol problems—derived from GWAS estimates in one population-based sample—would predict alcohol problems in adolescence in a second, independent, population-based sample; and (2) parenting and peer factors in adolescence would moderate polygenic risk to predict alcohol problems in the independent sample.

## 2. Experimental Section

We drew upon two population-based samples in the present study. GWAS results from the Avon Longitudinal Study of Parents and Children (ALSPAC) [23] were used to create polygenic risk scores in the independent FinnTwin12 sample [24]. The samples and measures are described in greater detail below. 

### 2.1. Avon Longitudinal Study of Parents and Children

The ALSPAC sample included 15,247 pregnancies from women residing in Avon, UK with expected dates of delivery between April 1991 and December 1992, resulting in 15,458 fetuses. Of this total sample of 15,458 fetuses, 14,775 were live births and 14,701 were alive at 1 year of age. Additional details regarding the sample can be found in Boyd *et al.* [25]. Ethical approval for the study was obtained from the ALSPAC Ethics and Law Committee and the Local Research Ethics Committees. In the present study, we used data from unrelated participants who completed an alcohol assessment at 16 and/or 18 years of age (5952 participants) for whom there were also genotypic data (n = 4304). Please note that the study website contains details of all the data that is available through a fully searchable data dictionary (http://www.bris.ac.uk/alspac/researchers/data-access/data-dictionary).

#### 2.1.1. Alcohol Problems Factor Score

We measured alcohol problems using a factor score that included ten items from the Alcohol Use Disorders Identification Test (AUDIT) [26], seven DSM-IV Alcohol Dependence criteria [27], and three additional measures related to alcohol problems (getting into fights, police involvement, and drinking to alleviate withdrawal symptoms) that were collected as part of the age 18 assessment. To increase our sample size, we also imputed age 18 alcohol problems data for the participants who completed the age 16 alcohol assessment, but not the age 18 assessment (n = 1993) using imputation software IVEware [28]. Frequency and correlation checks after imputation showed that all imputations kept similar frequency distributions and that imputed and original variables were closely correlated. The results of an exploratory factor analyses indicated one main factor (eigenvalue = 6.78) that broadly measured heavy alcohol use and problems. We then ran a confirmatory factor analysis to calculate factor scores using Mplus 6.11 [29]. All items’ factor loadings were >0.30, and the items with the greatest loadings were: frequency of heavy drinking (6 or more drinks on one occasion); drinks per day on drinking days; injuries as a result of drinking; and tolerance. In total, alcohol problems factor scores were calculated for 5952 participants.

#### 2.1.2. Genotyping

ALSPAC participants were genotyped from blood samples using the Illumina 550K custom chip (San Diego, CA, USA). Multi-dimensional scaling modeling seeded with HapMap Phase II release 22 reference populations was used to identify individuals of non-European descent. To reduce bias introduced by population stratification, individuals of non-European descent were removed from subsequent analyses. Those of European descent were imputed to HapMap Phase II (release 22, NCBI build 36, hg18) using the Markov Chain Haplotyping software (MACH v.1.0.16) [30]. SNPs that were in Hardy-Weinberg equilibrium (*p* > 5 × 10^−7^) with a final call rate of >95%, and minor allele frequency >1% were used in the imputation procedure. The 2,450,300 autosomal SNPs that exceeded an Rsq metric of 0.3 and had a minor allele frequency >1% following imputation were used in the GWAS. Additional, detailed GWAS data cleaning information for this sample are available in Fatemifar *et al.* [31]. 

### 2.2. FinnTwin12

Our second, independent sample was FinnTwin12 [24]—a population-based twin sample identified through Finland’s Population Register Center. Approximately 2700 pairs of twins were initially enrolled between ages 11–12 and have been contacted for multiple follow-up assessments of behavioral, emotional, and physical health. In the present study we used data from 1162 participants (467 MZ individuals, 684 DZ individuals, and 11 individuals of unknown zygosity; 53% female, 47% male) for whom there were genome-wide association (GWA) data. Relevant phenotypic data from a psychiatric interview and self-report measures of parental knowledge (n = 1115) and peer deviance (n = 1116) at age 14 were available for a subset of the GWA sample.

#### 2.2.1. Alcohol Problems, Parental Knowledge, and Peer Deviance

Alcohol problems, parental knowledge, and peer deviance were assessed at age 14. The alcohol measure was a sum score of alcohol problems (range 0–30) from the Child version of the Semi-Structured Assessment for the Genetics of Alcoholism [32]. Sample items included needing 50% more alcohol to get an effect, being unable to cut down, reducing important activities to drink, and experiencing withdrawal symptoms. 

The parental knowledge measure was the sum score of four adolescent self-report items adapted from Chassin and colleagues [33] about the degree to which their parents know about their daily plans, activities and whereabouts, how they spend their money, and where/who they are with when not at home. Responses were made on a 4-point scale ranging from *almost always* to *rarely or never*, and were summed such that high scores indicate low parental knowledge (more risk; range 4–16). 

The peer deviance measure was the sum score of four adolescent self-report items regarding the number of friends/acquaintances who drink, smoke, use drugs, and get into trouble at school. Responses were made on a 4-point scale ranging from *none* to *more than* *five*, and were summed such that high scores indicate high peer deviance (more risk; range 4–16). 

#### 2.2.2. Genotyping

Genome-wide data were collected using blood samples obtained at the age 22 assessment. Genotyping was performed at the Wellcome Trust Sanger Institute (Hinxton, UK) on the Human670-QuadCustom Illumina BeadChip (Illumina, Inc., San Diego, CA, USA), as previously described in Broms *et al.* [34]. The data were checked for minor allele frequency (MAF > 1%), genotyping success rate per SNP and per individual (>95%; >99% for SNPs with MAF < 5%), Hardy-Weinberg Equilibrium (HWE *p* > 1 × 10^−6^), sex, and heterozygosity. In addition, to check whether any individuals were unexpectedly related to each other, a multidimensional scaling plot (using a pairwise-IBS matrix) with only one member of each known family was created. After the pedigree was checked for accuracy, the basic filters (MAF, genotyping success, HWE) were reapplied to the data.

Imputation was performed by using ShapeIT [35] in pre-phasing and IMPUTE2 [36] for genotype imputation, with the 1000 Genomes Phase I integrated variant set release (v3) reference panel. The posterior probability threshold for “best-guess” imputed genotypes was 0.9. Genotypes below the threshold were set to missing. Genotypes for altogether 6,729,635 SNPs were available for analysis. 

### 2.3. Analytic Plan

#### 2.3.1. Genome-Wide Association Analysis in the ALSPAC Sample

The GWAS was conducted using MACH2QTL [37] and was limited to individuals of European descent. Sex was included as a covariate.

#### 2.3.2. Calculation of Polygenic Scores in FinnTwin12

We used ALSPAC GWAS estimates from the alcohol problems factor score to calculate polygenic scores for FinnTwin12 using the --score procedure in PLINK [38]. We computed a linear function of the number of score alleles an individual possessed weighted by the product of the sign of the SNP effect and the negative logarithm (base 10) of the associated GWAS *p*-value. This retains the same direction between calculated and original output values. Of the 2,450,300 autosomal SNPs that passed quality control in the ALSPAC sample, 2,221,783 (91%) were available in the FinnTwin12 sample. 

There are no set criteria for creating maximally informative polygenic scores [39], and so we created a series of scores using *p*-value thresholds ranging from 0.05 to 0.50. Table 1 summarizes the number of SNPs meeting each threshold in the ALSPAC sample, as well as the number and percent of those SNPs that were available in the FinnTwin12 sample. Previous work using polygenic approaches indicates that pruning for linkage disequilibrium (LD) does not substantially change the results [19,40]. In view of this, we chose to incorporate all SNPs meeting each polygenic threshold into our scores.

**Table 1:** Autosomal single nucleotide polymorphisms (SNPs) contributing to each polygenic threshold in Avon Longitudinal Study of Parents and Children (ALSPAC) sample, and availability in FinnTwin12.

Polygenic threshold	Number of autosomal SNPs meeting threshold in ALSPAC	Number (percent) of SNPs available in FinnTwin12
*p* ≤ 0.05	125,969	113,992 (90.5%)
*p* ≤ 0.10	250, 244	226,789 (90.6%)
*p* ≤ 0.20	495,760	449,273 (90.6%)
*p* ≤ 0.30	739,758	670,293 (90.6%)
*p* ≤ 0.40	984,167	891,782 (90.6%)
*p* ≤ 0.50	1,231,165	1,115,557 (90.6%)

#### 2.3.3. Polygenic Association and Moderation Analyses in FinnTwin12

We used partial Pearson correlations, controlling for sex, to test associations between the FinnTwin12 polygenic scores and alcohol problems. We used moderated multiple regression to test our gene-by-environment interaction hypotheses that parental knowledge and peer deviance would moderate the predictive association of polygenic scores with the age 14 alcohol problems measure. For these analyses, the parameters of interest were the statistical interactions between the environmental factors (parental knowledge and peer deviance) and the polygenic scores. The main effects of sex and the environmental factors were used as covariates in the relevant models. Parental knowledge, peer deviance, and polygenic scores were centered on their means prior to running moderation analyses to reduce co-linearity among predictor variables.

## 3. Results and Discussion

### 3.1. Descriptive Statistics and Zero-Order Correlations

Descriptive statistics for the focal variables and for an illustrative polygenic score (using the *p*≤ 0.05 threshold) are presented in Table 2. MZ twins’ alcohol problems were correlated at *r* = 0.53 (232 pairs; *p* < 0.01), and DZ twins were correlated at *r* = 0.36 (277 pairs; *p* < 0.01). This pattern of twin correlations suggests that additive genetic effects accounted for approximately 34% of the variance in alcohol problems. Lower parental knowledge (indexed by higher scores on the parental knowledge scale used here) and higher peer deviance were associated with higher levels of alcohol problems [*r*(1113) = 0.29 and *r*(1114) = 0.35, both *p-*values < 0.01, respectively], which is consistent with previous work indicating that more permissive and deviant environments are associated with a greater amount of adolescent substance use [33,41,42]. 

**Table 2:** FinnTwin12 descriptive statistics for focal study variables.

Variable	M	SD	Min	Max
Alcohol problems (age 14), range 0–30	0.29	0.96	0	8
Parental knowledge (age 14), range 4–16	6.62	2.08	4	15
Peer deviance (age 14), range 4–16	7.91	3.14	4	16
Polygenic score (*p*≤ 0.05 threshold)	−0.07	0.02	−0.13	0.00

Abbreviations: M, mean; SD, standard deviation; Min, minimum observed value; Max, maximum observed value.

### 3.2. Polygenic Associations with Alcohol Problems

**Figure 1:**
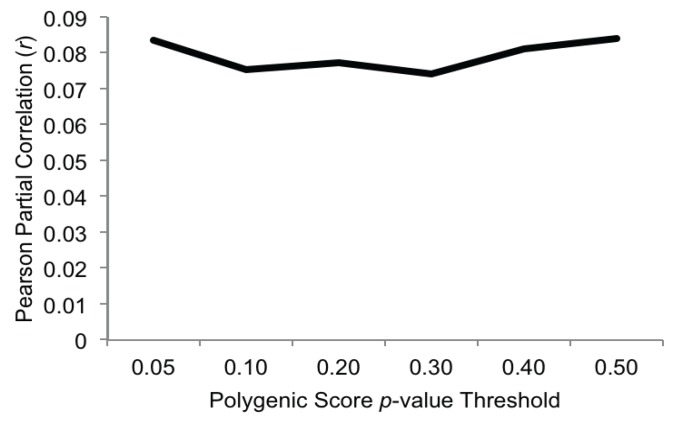
Pearson partial correlations (controlling for sex) between polygenic scores and age 14 alcohol problems (all *p-*values ≤ 0.01) in FinnTwin12 (n = 1161).

Partial correlations (controlling for sex) between the polygenic scores and alcohol problems are presented in Figure 1. As expected, higher polygenic scores predicted higher alcohol problems at age 14 (range of Pearson partial correlations 0.07–0.08, all *p-*values < 0.01). This is consistent with previous studies of other psychiatric conditions (such as bipolar disorder [19], schizophrenia [43] and externalizing disorders [40]) in showing that polygenic scores derived from GWAS weights from one sample can have predictive validity in an independent sample. Furthermore, our effect sizes were similar in magnitude to those observed in a polygenic analysis of a behavioral disinhibition measure (which included antisocial behavior, nicotine use/dependence, alcohol consumption and dependence, and drug use) [40]. 

The magnitude of the associations between polygenic scores and alcohol problems was fairly consistent across the range of selected *p*-value thresholds, and accounted for, on average, 0.63% of the variance in alcohol problems (range 0.55%–0.70%). To be sure that our effects were not driven by non-independence within the sample, we re-ran the association analyses after randomly dropping one member from each twin pair (n = 634) and found the same pattern of results. This is substantially lower than the estimate (derived from the pattern of MZ and DZ twin correlations in the same sample) that additive genetics effects account for 34% of the variance in alcohol problems. We note, however, that heritability estimates derived from twin models and the variance accounted for by a polygenic score are not directly comparable. Polygenic scores are composed of SNPs across a range of *p*-value thresholds, and thus their genetic informativeness is likely to be somewhere between a polygenic risk score based on genome-wide significant SNPs and SNP heritability as derived through methods that estimate the variance explained by genome-wide markers (e.g., GCTA; [44]). The limited amount of variance accounted for in our analyses may be attributable to the fact that GWAS-derived polygenic scores only account for common (*versus* rare; [45]) genetic variation; accordingly, incorporating rare genetic variation in polygenic scores may be an important direction for future research. In addition, the limited variance accounted for may also be attributable to the relatively small sample from which we derived our GWAS weights owing to the fact that smaller samples are likely to have a higher signal-to-noise ratio compared to larger samples.

We also tested whether there was evidence for gene-environment correlation by using Pearson correlations to examine the associations between polygenic scores and the parental knowledge and peer deviance environmental measures. As expected, higher polygenic scores were modestly associated with lower parental knowledge, although the effect was of a small magnitude and not significant [*r* (1113) = 0.05, *p* = 0.09]. Higher polygenic scores were also modestly associated with higher peer deviance [*r* (1114) = 0.08, *p* < 0.01]. This is consistent with previous evidence from twin studies showing that externalizing-spectrum behaviors such as alcohol use, tobacco use, and conduct problems are genetically correlated with environmental factors [7,8]. These findings highlight the complex interplay between genetic and “environmental” influences on behavioral outcomes such as alcohol problems [46].

### 3.3. Gene-by-Environment Interactions

The polygenic score using the *p* ≤ 0.05 threshold accounted for the greatest proportion of variance (0.70%) in age 14 alcohol problems, and we carried this score forward for the gene-by-environment analyses in view of earlier suggestions that SNPs having a nominal association with a phenotype are likely to be enriched for gene-by-environment interaction [47].

**Figure 2:**
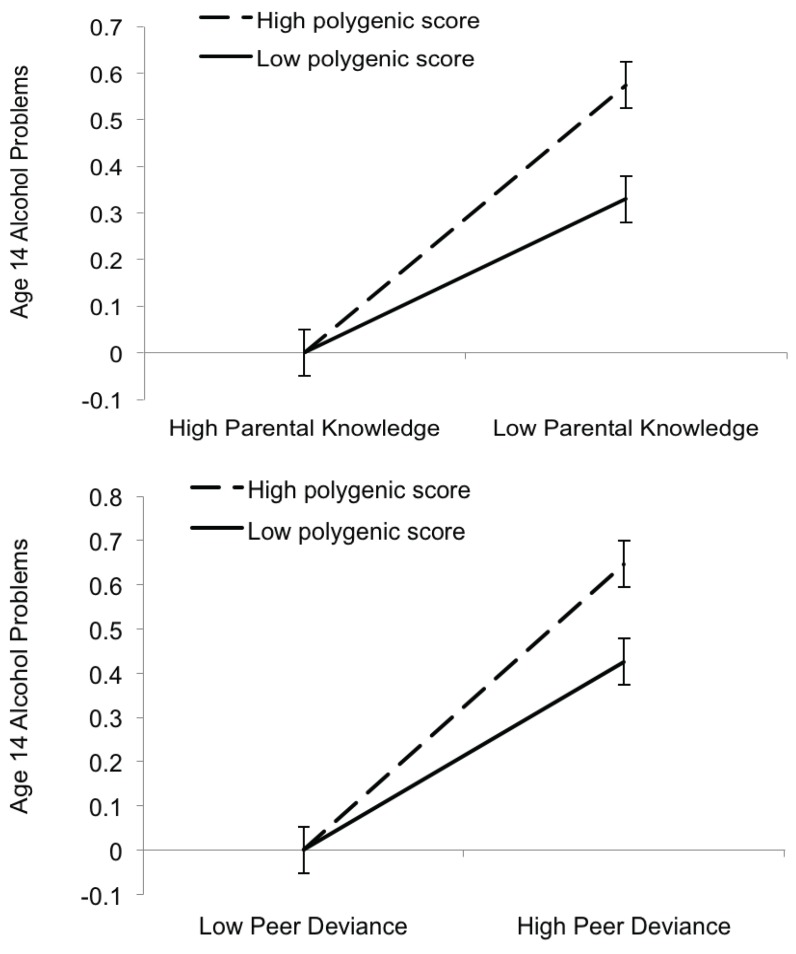
Parental knowledge (top) and peer deviance (bottom) moderate polygenic risk to predict age 14 alcohol problems in FinnTwin12. Interactions are plotted as predicted values based on the moderated multiple regression equation for age 14 alcohol problems. Illustrative low and high values (±1 SD of mean) for the polygenic scores, parental knowledge, and peer deviance are shown. The predicted values for high parental knowledge and low peer deviance were out of bounds (negative values) and were set to zero—the lowest possible value for the alcohol problems measure. Error bars are equal to the standard deviation of the model residuals divided by the square root of the sample size. We note that high scores on the parental knowledge scale indicate low parental knowledge (*i.e.*, more risk). For ease of interpretation, we have formatted the axis for each figure so that the riskier environment appears on the right.

Moderated multiple regression analyses indicated that parental knowledge and peer deviance moderated the associations of polygenic scores with age 14 alcohol problems (Table 3). Genetic influences were more pronounced under conditions of low parental knowledge or high peer deviance compared to conditions of high parental knowledge or high peer deviance (Figure 2). The interactions with parental knowledge and peer deviance accounted for 0.33% and 0.30% of the variance in alcohol problems, respectively. To verify that our effects were not driven by non-independence within the sample, we note that the same pattern of effects was found when we re-ran the moderation analyses after randomly dropping one member from each twin pair (n = 634).

**Table 3:** FinnTwin12 sample. Moderated multiple regression of age 14 alcohol problems on sex, polygenic score, parental knowledge, and the interaction of polygenic score and parental knowledge (top; n = 1115). Moderated multiple regression of age 14 alcohol problems on sex, polygenic score, peer deviance, and the interaction of polygenic score and peer deviance (bottom; n = 1116).

**Parental Knowledge**					
	*b*	*SE*	*t*	*P*	Δ*R*^2^
Intercept	***0.16***	***0.04***	***3.97***	**<*0.01***	**--**
Sex	***0.23***	***0.06***	***4.17***	**<*0.01***	***0.006***
Polygenic score	**3.10**	**1.40**	**2.21**	**0.03**	***0.006***
Parental knowledge	***0.14***	***0.01***	***10.31***	**<*0.01***	***0.088***
Polygenic score × Parental knowledge	**1.54**	**0.68**	**2.27**	**0.02**	**0.003**
**Peer Deviance**					
	*b*	*SE*	*t*	*P*	Δ*R*^2^
Intercept	***0.19***	***0.04***	***4.88***	**<*0.01***	**--**
Sex	***0.17***	***0.05***	***3.07***	**<*0.01***	***0.006***
Polygenic score	2.75	1.38	1.99	0.05	0.006
Peer deviance	***0.11***	***0.01***	***12.43***	**<*0.01***	***0.120***
Polygenic score × Peer deviance	**0.94**	**0.44**	**2.11**	**0.04**	**0.003**

Boldfaced statistics indicate *p* < 0.05. Boldfaced and italicized statistics indicate *p* < 0.01. Abbreviations: n = sample size, *b*, unstandardized regression estimates; *SE*, standard error for *b*; *t*, *t*-statistic; *P*, *p*-value; Δ*R*^2^, step-wise change in variance accounted for by each parameter in model.

Although the effect sizes for the polygenic score X environment interactions were small, the pattern of effects is consistent with previous findings from the twin literature. Multiple independent twin studies find that parenting and peer environmental factors moderate latent genetic influences for alcohol use and related outcomes such that genetic influences increase under conditions of low parental knowledge and high peer deviance [6,7,8,9,48]. The convergence between the pattern of gene-environment interactions from twin studies and measured polygenic effects is encouraging, and suggests that polygenic approaches may be a useful way to characterize gene-environment interplay for aggregate genetic risk using measured genotypic data. 

In addition to these core analyses, we ran a series of supplementary analyses to examine the robustness of our effects after controlling for gene-environment correlation and after transforming our alcohol problems dependent variable to a logarithmic scale. Gene-environment correlation can produce spurious gene-environment interaction effects; likewise, interaction effects are known to be sensitive to scale. Accordingly, our supplementary analyses were intended to address concerns that our observed gene-environment interaction effects could be statistical artifacts.

To control for gene-environment correlation in our parental knowledge analyses, we used residualized polygenic score and parental knowledge variables in our model. To calculate residualized variables, we regressed polygenic scores onto parental knowledge (and *vice versa*) and saved the residuals for use in the moderation models. Using residualized variables in this way statistically eliminates gene-environment correlation from the model because the genetic and environmental effects have been partialled from one another. We used the same method to calculate residualized polygenic score and peer deviance variables for our peer deviance analyses. The moderation effect for parental knowledge continued to be statistically significant; however, the moderation effect for peer deviance trended in the same direction but was not statistically significant (unstandardized regression coefficients (*b*) and *p*-values (*p*) for interaction terms: *b* = 1.33, *p* = 0.05 and *b* = 0.66, *p* = 0.14, respectively) when we used residualized values in our analyses. 

To test whether our interaction effects could be attributed to the scale of the alcohol problems measure, we used a log-transformed version of the measure (*i.e.*, log_10_ (alcohol problems + 1)) in our analyses. The interaction effects trended in the expected direction for parental knowledge and peer deviance, albeit failing to reach significance (unstandardized regression coefficients (*b*) and *p*-values (*p*) for interaction terms: *b* = 0.51, *p* = 0.07 and *b* = 0.30, *p* = 0.10, respectively). As a set, these supplementary analyses demonstrate that the moderation effects were modestly attenuated after controlling for gene-environment correlation and changing the scale of the alcohol problems outcome variable, but they continued to trend in the same direction and did not entirely go away. 

Although the causal relationships among the genetic and environmental variables examined here are unknown, we note that early findings from genetically-informed randomized prevention studies suggest that efforts aimed at reducing environmental risk factors for adolescent alcohol use and related behavior problems may be particularly effective for those who are genetically predisposed toward developing such problems. For example, adolescents with either the short/short or short/long genotype of *SCL6A4(5-HTT)* who took part in a family-based prevention-intervention program aimed at increasing family cohesion were less likely to initiate risk behaviors (alcohol use, marijuana use, and sex) across a 29-month period compared to their counterparts in the control condition [49]. Examining whether efforts to bolster parental knowledge or reduce peer deviance attenuate polygenic risk for alcohol problems is an important direction for future research.

### 3.4. Set-Based Analyses Examining Enrichment for Gene-Environment Interaction among Top SNPs

The polygenic analyses indicated significant gene-environment interaction effects with parental knowledge and peer deviance, and we used set-based analyses to probe whether the individual top SNPs contributing to our polygenic scores were themselves enriched for gene-environment interaction. We examined this question using the set of top SNPs (*p* ≤ 0.0001) from the ALSPAC GWAS. We selected this relatively stringent *p*-value threshold in view of the computing resources required to perform the permutation analyses described below. Of the 311 SNPs meeting this threshold in ALSPAC, 279 (90%) were available in FinnTwin12. We pruned by LD in the FinnTwin12 sample in order to reduce the set to include only independent (*r*^2^ < 0.50) SNPs, which resulted in 76 SNPs. Because LD calculations should be made on independent individuals, we used a randomly-selected sample of independent individuals in the FinnTwin12 sample (*n* = 634) for this purpose. We then permuted the phenotypic and covariate information for these individuals 100,000 times while keeping the genotypic information (LD) unchanged. For each of these permuted datasets, we examined gene-environment interaction effects for parental knowledge and peer deviance. To calculate empirical *p*-values, we used the equation (R + 1)/(N + 1). R is the number of permutations where the sum of the absolute value of the *t*-scores for significant SNP interaction effects (*p* < 0.05) exceeded the sum of the absolute value of the *t*-scores for significant SNP interaction effects in the observed data. N is the number of permutations (100,000).

Our empirical *p*-values were 0.32 and 0.71 for parental knowledge and peer deviance, respectively. This indicates that the SNPs contributing to our polygenic scores were not individually enriched for gene-environment interaction, and further suggests that the polygenic moderation effects that we observed occur at the aggregate genetic level rather than at the level of individual SNPs. Attempts to replicate these effects in other independent datasets are critical for better understanding the contributions of individual SNPs to the aggregate effects observed for polygenic scores.

### 3.5. Limitations

Our results should be interpreted in the context of their limitations. First, the participants in our two samples were of European descent, the latter exclusively of Finnish descent, which may limit the generalizability of the present findings to samples from the same ancestral background. Second, although our association and moderation findings were in the expected direction, the effect sizes were quite small—often accounting for less than 1% of the variance. Although there is much enthusiasm for personalized medicine approaches [50] that use genome-wide information to identify for whom and under what conditions prevention and intervention efforts are likely to be effective, our results caution against using empirically-derived GWAS scores in a clinical setting for complex behavioral outcomes such as alcohol problems due to the fact that they account for a limited proportion of the variance [51]. Third, alcohol problems in FinnTwin12 were assessed at age 14. Accordingly, endorsements of alcohol problems at this age may represent a more severe phenotype than those at age 18 in ALSPAC. The age and measurement differences across the ALSPAC and FinnTwin12 samples may explain, in part, the low percentage of variance accounted for by the polygenic score. Finally, the polygenic approach adopted here is limited in that it does not attempt to implicate the specific genes involved in alcohol problems. Additional methods, such as gene set approaches that examine whether SNPs included in a polygenic score are located in functionally related genes [52], are well suited to identify the potential biological mechanisms underlying polygenic effects. 

## 4. Conclusions

Higher polygenic predispositions for alcohol problems (based on GWAS estimates from a population-based sample of young adults) predicted a higher number of adolescent alcohol problems in an independent, population-based sample. In addition, environmental factors in adolescence moderated these polygenic predispositions. Genetic predispositions were more important under conditions of low parental knowledge and high peer deviance. These gene-by-environment interactions, although small in magnitude, are consistent with previous findings from studies that show that environments low in social control or high in social opportunity permit the expression of genetic predispositions [10]. In contrast, environments high in social control or low in social opportunity may inhibit the expression of that same predisposition. Accordingly, prevention and intervention efforts that increase parental knowledge and decrease affiliations with deviant peers may be one strategy for reducing risk for adolescents with genetic predispositions toward alcohol problems; however additional study is needed before making strong claims about the potential effectiveness of such interventions. 
